# Developmental Trajectory of Inattention and Its Association With Depressive Symptoms in Adolescence: Peer Relationships as a Mediator

**DOI:** 10.3389/fpsyg.2021.736840

**Published:** 2022-02-01

**Authors:** Sohee Park, Hyein Chang

**Affiliations:** Department of Psychology, Sungkyunkwan University, Seoul, South Korea

**Keywords:** inattention, peer relationship, depression, adolescence, Korean Children & Youth Panel Survey 2010 (KCYPS 2010)

## Abstract

This study investigated the developmental trajectory of inattention symptoms as a predictor of later depressive symptoms in adolescence, and examined potential mediating role of peer relationships in this process. Participants were adolescents who were part of the large longitudinal panel study on Korean Youths, Korean Children & Youth Panel Survey 2010 (KCYPS 2010) of the National Youth Policy Institute (NYPI). Specifically, data were drawn from two cohorts of KCYPS that differed in participant age (Panel 1: 2003 birth cohort, *n* = 2,342, 48.2% girls; Panel 2: 2000 birth cohorts, *n* = 2,378, 40.0% girls). We analyzed data collected from 2010 to 2016 when children in panel 1 were 6–7 to 12–13 years old, and children in panel 2 were 9–10 to 15–16 years old. Results of latent growth modeling (LGM) were as follows. In Panel 1, the inattention symptoms increased from 9–10 to 12–13 years. Otherwise, the inattention symptoms decreased from 11–12 to 15–16 years in panel 2. Additionally, in both panels, initial status and slope of inattention significantly predicted later levels of depressive symptoms, and peer relationships partly mediated the association between inattention trajectory and depressive symptoms. The findings are discussed with respect to considering the growth of inattention and the quality of peer relationships as promising targets for early identification and intervention of depression in adolescents.

## Introduction

Attention-Deficit Hyperactivity Disorder (ADHD) is one of the most common neurodevelopmental disorders affecting about 5% of children and adolescents, with boys at higher risk for developing ADHD than girls (Polanczyk et al., 2007; American Psychiatric Association [APA] and DSM-5 Task Force, 2013). Children with ADHD not only demonstrate primary symptoms of the disorder, inattention, hyperactivity, and impulsivity, but also suffer from various functional problems, including poor academic performance, parent-child relationship problems, and difficulties in peer relationships (Hoza et al., 2005b; Ostrander et al., 2006; Daley and Birchwood, 2010; Humphreys et al., 2013). Children with ADHD are also at increased risk for developing comorbid psychiatric disorders (Mannuzza and Klein, 2000). Furthermore, approximately 50% of children with ADHD may still meet diagnostic criteria for ADHD in adulthood (Lara et al., 2009), who may continue to struggle with psychological problems such as depression, anxiety, and personality disorders, as well as other difficulties such as lower college entrance rates and higher crime rates in adulthood (Mannuzza and Klein, 2000). Therefore, elucidating mechanisms by which ADHD symptoms may lead to socioemotional difficulties beyond childhood may be crucial for early prediction and prevention of problems accompanying ADHD.

Attention-deficit hyperactivity disorder is composed of two types of symptoms, inattention and hyperactivity-impulsivity (American Psychiatric Association [APA] and DSM-5 Task Force, 2013). Although both symptoms appear at all ages, they seem to follow different trajectories as children get older (Franke et al., 2018). Specifically, many studies have demonstrated that hyperactivity-impulsivity symptoms are more salient at young ages which tend to decline over time, while inattention symptoms become more apparent beginning middle childhood and are more persistent into adolescence and adulthood (Biederman et al., 2000; Willcutt et al., 2012; Franke et al., 2018). Indeed, a longitudinal study has found that the proportion of ADHD inattention subtype has shown to increase from 37 to 64% in adolescence, whereas hyperactivity-impulsivity and combined subtype has shown to decrease from 20 to 8%, and 43 to 28%, respectively (Hurtig et al., 2007). Moreover, inattention symptoms remained stable from early childhood to late adolescence contrary to hyperactivity-impulsivity symptoms which decreased by more than half over time (Holbrook et al., 2016). Therefore, it has been suggested that inattention symptoms may be particularly important to consider as a contributor to socioemotional adjustment in adolescence and young adulthood period (Biederman et al., 2000; Molina et al., 2009).

However, most previous studies on ADHD have focused on childhood, and much less research has been conducted on how ADHD symptoms may continue into adolescence. In particular, very few studies have investigated the developmental trajectory of inattention and the existent studies have yielded mixed findings. Some studies that have encompassed childhood and adolescence have found that both hyperactivity-impulsivity and inattention decrease over time. For example, studies that traced ADHD symptoms from 7 to 19 years, from 8 to 16 years, and from 8 to 14 years have found decreasing trajectories of inattention (Döpfner et al., 2015; Pingault et al., 2015; Larsen et al., 2020). Other studies have found that inattention symptoms may remain constant throughout middle childhood and adolescence (Jester et al., 2008; Salla et al., 2016). Lastly, although relatively few, studies have also reported increasing trajectories. For instance, inattention symptoms have shown to increase from 8 to 17 years (Larsson et al., 2011), and from 6 to 12 years (Pingault et al., 2014). Together, in contrast to hyperactivity-impulsivity, inattention symptoms of ADHD may continue to fluctuate into adolescence, although its specific trajectory is yet to be clarified.

In addition to their difficulties in attention and behavior regulation, children with ADHD suffer functional impairment in several domains such as academic and psychosocial adjustment (Mannuzza and Klein, 2000; Biederman et al., 2008; Daley and Birchwood, 2010; Rajendran et al., 2013; Bunford et al., 2018). Moreover, individuals with ADHD may develop psychiatric disorders such that, depending on sample, about 40–80% of children and adolescents with ADHD have been shown to be diagnosed with comorbid disorder(s) (Gillberg et al., 2004; Elia et al., 2008; Larson et al., 2011). In the current study, we focus on elucidating mechanisms that link ADHD and depression.

Depression occurs in youths with ADHD at a greater rate than those without ADHD (Blackman et al., 2005; Daviss, 2008). Additionally, ADHD patients who are also depressed have been found to demonstrate worse prognosis over time (Blackman et al., 2005; Biederman et al., 2008). For example, in a longitudinal study, adolescents with ADHD had 5.1 times higher risk for depression, earlier age at onset, longer duration of depressive episodes, and higher rate of suicidality and hospitalization compared with control groups (Biederman et al., 2008). Generally, the onset of depression is later than ADHD (Kovacs et al., 1994; Biederman et al., 1995). The most salient period of vulnerability to depression is known to be adolescence such that the mean age of onset of Major Depressive Disorder is known to be 14.9 years among adolescents, and the probability of experiencing a depressive episode increases dramatically after about 14 years of age (Lewinsohn et al., 1998; Ostrander et al., 2006). Thus, understanding how ADHD may operate as a risk factor for depression would be useful for effective intervention of adolescent depression.

Attention-deficit hyperactivity disorder symptoms may contribute to depression *via* a number of pathways. Specifically, cumulative effects of impairments accompanied with ADHD may function as stressors that may lead youths with ADHD to become depressed (Waxmonsky, 2003; Blackman et al., 2005; Ostrander et al., 2006; Herman et al., 2007). Particularly, in this study, we focus on peer relationships as a processible mediator of the relationship between inattention and depression. Previous studies have shown that inattentive individuals may demonstrate poor social competence and isolation in peer contexts (Maedgen and Carlson, 2000; Hoza et al., 2005b; Shaw-Zirt et al., 2005). These children tend to be more timid, socially withdrawn, and less participating in social interaction with their peers (Wheeler and Carlson, 1994). They also typically show lower levels of prosocial behaviors compared to similar age children without inattention symptoms (Tseng et al., 2012). Their lack of social competence may be linked to cognitive deficiencies associated with inattention symptoms. Previous studies have shown that neurological and executive function deficits that accompany inattention symptoms may also interfere with social cognition such as facial recognition and theory of mind that are critical for successful functioning in social interaction (Shaw-Zirt et al., 2005; Ibáñez et al., 2011; Tseng and Gau, 2013; Hilton et al., 2017). In addition, inattention symptoms may interrupt youths from actively participating in social interaction by disturbing them from acquiring rules and behaviors, and making appropriate social decisions (Cunningham et al., 1985; Mrug et al., 2007).

Difficulties in peer relationships may serve as a robust risk factor for depression, especially in adolescence. Peers form a relational context in which adolescents primarily seek emotional support such as a sense of security and belongingness (Chen et al., 2012). In particular, compared to other age groups, adolescents spend more time with their peers than with their families, and exert a lot of energy on peer relationships (Larson et al., 1996; Field et al., 2002). Thus, if adolescents are rejected or isolated from peers during this period, they may feel distressed and frustrated, and this in turn may contribute to an increased risk for depression (Beck, 1987; Cole and Carpentieri, 1990). Moreover, the construction and maintenance of healthy social relationships have significant developmental meaning in adolescence (Erikson, 1993), and negative experiences in peer context may contribute to the development of negative cognitions about self and others, and expectations about future interpersonal interaction (Rudolph et al., 1997; Ladd and Troop-Gordon, 2003). Indeed, compromised quality of peer relationships has been recognized as one of the strongest predictors of depression in many studies with children and adolescents (Bernaras et al., 2013; Platt et al., 2013; Garaigordobil et al., 2017). For instance, a longitudinal study found that children who are less preferred by peers tend to have more depressive symptoms, and loneliness mediated this relationship (Fontaine et al., 2009). Furthermore, studies with children in Asian cultures have also shown similar results. For example, in a study with Chinese children, peer relationships negatively predicted later depression (Chen et al., 2012). A study with Korean late school-age children also documented that the quality of children’s peer relationships significantly influenced their affective problems (Bang et al., 2018). In a systemic review, ADHD symptoms were found to form a diathesis for bullying, and bullying may function as a moderator or mediator in the association between ADHD and depression (Simmons and Antshel, 2021). Together, children who display inattention symptoms may experience difficulties in peer relationships, which in turn may contribute to their depressive symptoms.

However, very few studies have investigated the role of peer relationships in the association between inattention and depression in childhood (Ostrander et al., 2006; Humphreys et al., 2013), and none has examined such process in adolescents. Furthermore, despite prior findings that inattention symptoms may change with growth in childhood adolescence, no study to date incorporated changes in inattention as a predictor of peer relationships and/or depression. Thus, in this study, we aimed to refine the relationship between inattention trajectory, peer relationships, and depressive symptoms in adolescents by modeling developmental trajectory of inattention symptoms, and analyzing whether the identified trajectory of inattention predicts future depressive symptoms *via* its effect on peer relationships. We aimed to address those questions using two large-scale datasets of Korean adolescents.

## Materials and Methods

### Participants and Procedures

Participants were part of the Korean Children & Youth Panel Survey 2010 (KCYPS 2010) by the National Youth Policy Institute (NYPI) in South Korea^1^. This is a large-scale longitudinal survey on the development of Korean children and adolescents. KCYPS annually traced three different panel cohorts for 7 years from 2010 to 2016 that were selected based on a stratified multi-stage clustering sampling method. Data were collected using self-report questionnaires for adolescents and their parents.

For the purposes of this study, we used data from two panels. Panel 1 (2003 birth cohort) included 2,342 participants (boys = 1,211, girls = 1,131) who were 6–7 years old (First grade in elementary school in Korea) at the baseline (T1). Panel 2 (2000 birth cohort) included 2,378 participants (boys = 1,245, girls = 1,133) who were 9–10 years old (Fourth grade in elementary school) at the baseline (T1). For Panel 1, of the initial sample of 2,342 participants, 90.4, 88.3, and 85.5% participated in the study at T4, T6, and T7, respectively. For Panel 2, of the initial sample of 2,378 participants, 93.3, 84.8, 86.7, and 83.2% participated at T3, T5, T6, and T7, respectively. Selective attrition analysis revealed that, in both panels, no significant differences existed between families who stayed versus dropped out of the study over time in terms of child sex, parent education, family income, inattention, depressive symptoms, and peer relationships measured at the baseline.

### Measures

#### Inattention Symptoms

Levels of inattention symptoms were assessed using the seven-item Inattention subscale of the Emotional and Behavioral Problems Scale (Jo and Lim, 2003). Each item (e.g., “I often lose supplies such as pencils and erasers,” “Even if I am praised or punished, I will soon be distracted again”) was rated on a scale of 1–4, with lower scores indicating higher levels of inattention. In this study, we reverse coded the scores such that higher scores correspond to higher levels of inattention. In Panel 1, inattention symptoms were measured *via* adolescent self-report at T4 (9–10 years), T6 (11–12 years), and T7 (12–13 years). In Panel 2, adolescents reported on their inattention symptoms at T3 (11–12 years), T5 (13–14 years), T6 (14–15 years), and T7 (15–16 years). The Cronbach’s αs ranged from 0.805 to 0.819, all *p*s < 0.001, in Panel 1, and from 0.807 to 0.828, all *p*s < 0.001 in Panel 2.

#### Depressive Symptoms

Adolescents’ depressive symptoms were measured using the Korean version of the Symptom Checklist 90 Revised. This measure was originally developed by Derogatis et al. (1973), and was translated and validated in Korean by Kim et al. (1978). In KCYPS, the checklist was constructed for 4-score Likert scale, and among 13 questions for depression, 10 questions were used. In the current study, the higher score means the more severe depressive symptoms and we only used T7 data. The Cronbach’s α was 0.899 in Panel 1, and 0.893 in Panel 2.

#### Peer Relationships

At T7, adolescents rated their peer relationships using the Korean version of the Inventory of Parent and Peer Attachment (IPPA; Armsden and Greenberg, 1987; Kim, 1995). This nine-item inventory was consisted of three subscales of communication, trust, and isolation. Each item was rated on a 4-point scale, with higher scores indicating higher levels of each construct. In the current study, we reverse coded peer isolation scores such that the total scores are all in the direction of higher scores indicating higher quality of peer relationships. The Cronbach’s α was 0.817 in Panel 1 and 0.833 in Panel 2.

### Analytic Plan

Descriptive statistics and bivariate correlations were analyzed using SPSS 23.0. Subsequently, we employed latent growth modeling (LGM) in Mplus 7.0. LGM is special type of structural equation modeling designed to analyze temporal changes in longitudinal data (Byrne et al., 2008). In LGM, latent factors of intercept and slope are estimated to model the given data that has been collected at least across three time points (Duncan et al., 2006). Generally, LGM is implemented in two steps (Duncan and Duncan, 2004). In the first step (unconditional LGM), the best fitting growth curve for the given longitudinal data is identified. In the second step (conditional LGM), other exogenous and/or outcome variables are added to the unconditional model. In this study, for each Panel, we identified the best unconditional model of inattention, and then analyzed conditional model with peer relationships and depressive symptoms as mediator and outcome, respectively. Indirect effects were tested using the bootstrapping procedure (Shrout and Bolger, 2002). Multiple fit indices were considered in analyses. Specifically, *x*^2^ likelihood ratio statistic, root mean square error of approximation (RMSEA), Standardized Root Mean Square Residual (SRMR), Bentler’s normed comparative fit index (CFI), and Tucker Lewis Index (TLI). For missing data, the full-information maximum likelihood estimation (Muthén and Muthén, 2017) was used which accommodates missing data by using all available data based on the full sample (Enders and Bandalos, 2001).

## Results

### Preliminary Analyses

Descriptive statistics and bivariate correlations for Panel 1 and Panel 2 are presented in Tables 1, 2, respectively. The results were largely similar for Panel 1 and Panel 2 in terms of means of each variable as well as the magnitude and significance of correlations between the variables. However, inattention symptoms seemed to increase over time in Panel 1 whereas they seemed to decrease over time in Panel 2. In addition, the results of the *t*-tests are presented in Tables 3, 4. The results indicated that boys demonstrated higher levels of inattentive symptoms than girls, whereas girls showed higher levels of depressive symptoms than boys in both panels, with an exception of T7 inattention in Panel 2. Girls also reported higher levels of peer relationships quality than boys in both panels.

**TABLE 1:** Descriptive statistics and bivariate correlations (Panel 1).

	M	SD	1	2	3	4	5
1 Child sex	–	–	–				
2 Inattention, age 9–10	13.08	3.96	−0.175**	–			
3 Inattention, age 11–12	14.41	3.99	−0.123**	0.462**	–		
4 Inattention, age 12–13	14.79	3.95	−0.063**	0.355**	0.500**	–	
5 Depression, age 12–13	16.76	5.52	0.097**	0.184**	0.272**	0.419**	–
6 Peer relationships, age 12–13	28.44	4.19	0.133**	−0.180**	−0.217**	−0.272**	−0.421**

For child sex, 0 = boys, 1 = girls. **p < 0.01.

**TABLE 2:** Descriptive statistics and bivariate correlations (Panel 2).

	M	SD	1	2	3	4	5	6
1 Child sex	–	–	–					
2 Inattention, age 11–12	16.07	4.22	−0.118**	–				
3 Inattention, age 13–14	15.42	3.76	−0.049**	0.372**	–			
4 Inattention, age 14–15	15.22	3.80	−0.050**	0.321**	0.543**	–		
5 Inattention, age 15–16	14.65	3.69	−0.009	0.315**	0.474**	0.536**	–	
6 Depression, age 15–16	17.85	5.50	0.177**	0.125**	0.244**	0.250**	0.425**	–
7 Peer relationships, age 15–16	28.37	3.99	0.065**	−0.147**	−0.178**	−0.181**	−0.279**	−0.431**

For child sex, 0 = boys, 1 = girls. **p < 0.01.

**TABLE 3:** Results of *t*-test about sex difference (Panel 1).

	Mean (SD)		*t*	*df*
	Girls	Boys		
1 Inattention, age 9–10	12.37	13.75	8.19**	2115.54
2 Inattention, age 11–12	13.90	14.88	5.66**	2063.88
3 Inattention, age 12–13	14.53	15.03	2.84**	2,000
4 Depression, age 12–13	17.32	16.24	−4.38**	2,000
5 Peer relationships, age 12–13	29.01	27.90	−5.99**	2,000

For child sex, 0 = boys, 1 = girls. **p < 0.01.

**TABLE 4:** Results of *t*-test about sex difference (Panel 2).

	Mean (SD)		*t*	*df*
	Girls	Boys		
1 Inattention, age 11–12	15.54	16.54	5.639**	2212.86
2 Inattention, age 13–14	15.23	15.60	2.222**	2,068
3 Inattention, age 14–15	15.02	15.40	2.307*	2058.95
4 Inattention, age 15–16	14.61	14.68	0.392	1,977
5 Depression, age 15–16	18.87	16.92	−2.874**	1,977
6 Peer relationships, age 15–16	28.64	28.13	−2.875**	1,977

For child sex, 0 = boys, 1 = girls. *p < 0.05 and **p < 0.01.

### Latent Growth Modeling

#### Panel 1

##### Unconditional Model

The unconditional model for inattention symptoms measured at 9–10, 11–12, and 12–13 years in Panel 1 demonstrated adequate fit to the data: *x*^2^(1) = 5.773, CFI = 0.996, TLI = 0.987, RMSEA = 0.047, and SRMR = 0.013. The mean intercept and slope of inattention were 13.106 and 0.589 (both *p*s < 0.001), and the variance of intercept and slope were also significant (intercept = 10.740, *p* < 0.001; slope = 0.967, *p* < 0.001).

##### Conditional Model

**FIGURE 1:**
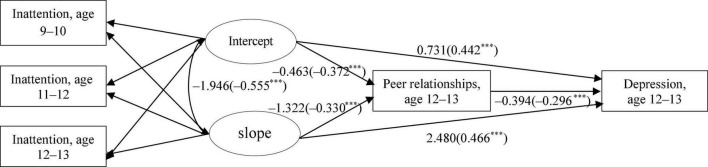
Results of latent growth modeling (LGM) (Panel 1). Peer relationship at age 12–13 and depressive symptoms at age 12–13 were controlled for child sex. Parameters in parentheses are standardized coefficients. ***p < 0.001.

The conditional model that incorporated child sex, peer relationships, and depressive symptoms to the unconditional model showed adequate fit: *x*^2^(6) = 87.756, CFI = 0.961, TLI = 0.903, RMSEA = 0.079, and SRMR = 0.050. As presented in Figure 1, the intercept and slope of inattention both significantly predicted peer relationships at 12–13 years (for intercept: *b* = −0.463, β −0.372, SE = 0.041, and *p* < 0.001; for slope: *b* = −1.322, β −0.330, SE = 0.169, and *p* < 0.001), which in turn was significantly associated with depressive symptoms at 12–13 years (*b* = −0.394, β −0.296, SE = 0.032, and *p* < 0.001). The results indicated that children who show lower levels of inattention and lower levels of increases in inattention over time were more likely demonstrated higher levels of peer relationships which in turn was associated with lower levels of depressive symptoms. Direct and indirect effects were tested using Bootstrapping. Specifically, indirect effects of inattention trajectory on depressive symptoms *via* peer relationships were significant for both the intercept (*b* = 0.182, β 0.110, SE = 0.020, and *p* < 0.001) and slope (*b* = 0.521, β 0.098, SE = 0.013, and *p* < 0.001). Additionally, direct effects of attention trajectory on depressive symptoms without peer relationships as a mediator was also significant for both the intercept (*b* = 0.731, β 0.442, SE = 0.056, and *p* < 0.001) and slope (*b* = 2.480, β 0.466, SE = 0.256, and *p* < 0.001).

#### Panel 2

##### Unconditional Model

The unconditional model for inattention symptoms measured at 11–12, 12–13, 14–15, and 15–16 years in Panel 2 demonstrated adequate fit to the data: *x*^2^(5) = 42.806, CFI = 0.980, TLI = 0.976, RMSEA = 0.058, and SRMR = 0.041. The mean intercept and slope of inattention were 16.114 and −0.344 (both *p*s < 0.001), and the variance of intercept and slope were also significant (intercept = 6.653, *p* < 0.001; slope = 0.270, *p* < 0.001).

##### Conditional Model

**FIGURE 2:**
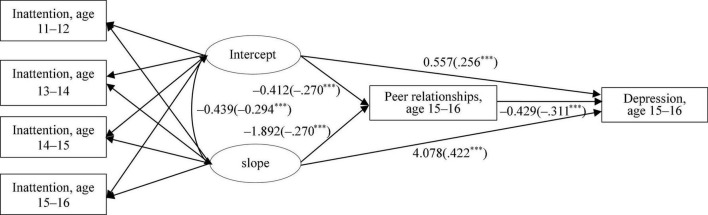
Results of latent growth modeling (LGM) (Panel 2). Peer relationship at age 15–16 and depressive symptoms at age 15–16 were controlled for child sex. Parameters in parentheses are standardized coefficients. ***p < 0.001.

The conditional model that incorporated child sex, peer relationships, and depressive symptoms to the unconditional model showed adequate fit: *x*^2^(13) = 117.692, CFI = 0.963, TLI = 0.941, RMSEA = 0.060, and SRMR = 0.035. As presented in Figure 2, the intercept and slope of inattention both significantly predicted peer relationships at 15–16 years (for intercept: *b* = −0.412, β −0.270, SE = 0.053, and *p* < 0.001; for slope: *b* = −1.892, β −0.270, SE = 0.404, and *p* < 0.001), which in turn was significantly associated with depressive symptoms at 15–16 years (*b* = −0.429, β −0.311, SE = 0.037, and *p* < 0.001). The results indicated that children who show lower levels of inattention and higher levels of decreases in inattention over time were more likely demonstrated higher levels of peer relationships which in turn was associated with lower levels of depressive symptoms. According to the result of Bootstrapping, indirect effects of inattention trajectory on depressive symptoms *via* peer relationships were significant for both the intercept (*b* = 0.177, β 0.084, SE = 0.028, and *p* < 0.001) and slope (*b* = 0.812, β 0.084, SE = 0.136, and *p* < 0.001). Additionally, direct effects of attention trajectory on depressive symptoms without peer relationships as a mediator was also significant for both the intercept (*b* = 0.557, β 0.265, SE = 0.076, and *p* < 0.001) and slope (*b* = 4.078, β 0.422, SE = 0.856, and *p* < 0.001).

## Discussion

The goals of this study were to examine the developmental trajectory of adolescents’ inattention symptoms and to examine mechanisms by which inattention trajectory may become associated with later depressive symptoms *via* its effect on peer relationships. We analyzed data from two birth cohorts from a large longitudinal study in South Korea.

An intriguing finding of our study is that the trajectories of inattention symptoms appear to follow opposite directions such that inattention symptoms may increase across middle childhood to early adolescence (Panel 1) and then may decrease across early to middle adolescence (Panel 2). We cannot make a direct comparison of the two datasets as they consist of different cohort of participants, but data collection procedures were quite similar in terms of study design and measures. Seemingly inconsistent results regarding developmental changes of inattention over time may have been partly caused by tracing different age groups. The results of this study based on two panel datasets that cover middle childhood and adolescence suggested that inattention symptoms may follow a quadratic, “Inverted-U” shaped trajectory which peaks approximately at the age of 12–13 years. This may be a significant period of development in relation to ADHD, particularly inattention symptoms. First, studies of human brain have demonstrated that cognitive processes related to attention such as planning, working memory, and executive function reach plateau of maturity at about 12–13 years accompanied by development of the prefrontal cortex and relative brain regions (Tipper et al., 1989; Giedd et al., 1999; Fuster, 2008; Uytun, 2018). Thus, cognitive components and brain regions associated with inattention symptoms typically mature around the age of 12 years. Additionally, children transfer from elementary school to middle school at 12 years in South Korea. It has been suggested that the transition to middle school may exacerbate the symptom presentation of inattention (Langberg et al., 2008). This is because significant environmental changes often accompany the transition from elementary to middle school (Eccles et al., 1997). For example, youths are required more cognitive and functional competences such as independence, self-control, and time management in middle school than elementary school, with increased demand for academic achievement (DuPaul and Stoner, 2014). Thus, it may be possible that the peak of inattention at 12–13 years in this study may reflect heightened stress in this transition.

However, the increasing trajectory of inattention observed in Panel 1 may be difficult to explain. The finding is inconsistent with prior studies that have found decreasing or stable trajectories of inattention across similar age periods (Salla et al., 2016; Larsen et al., 2020). We speculate that this may be due to the difference in how inattention was measured in current versus previous studies. Specifically, while prior studies measured inattention symptoms using parent or teacher reports (Salla et al., 2016; Larsen et al., 2020), we asked children to self-report their inattention symptoms. In contrast to hyperactive behavior, we thought that children or adolescent themselves may be valuable informants of their own inattention symptoms because inattention may be more difficult to observe by other people. However, children of different age may be more or less optimal reporters of their own behavior. As mentioned earlier, because children’s advanced cognitive functions develop rapidly during middle childhood and adolescence (Tipper et al., 1989; Giedd et al., 1999; Fuster, 2008; Uytun, 2018), including their ability to self-monitor their own behavior, they may have become more able to be aware of and recall their inattention as they mature with age.

Our findings and plausible explanations need to be confirmed using a single sample that is followed over years of time, because a major limitation of our speculation is that we combined two datasets from distinct cohorts. Moreover, given that this study traced community samples composed of mostly normally developing youths, inattention trajectory may not be identical in clinical populations. Therefore, it would be beneficial for future research to investigate how inattention changes over time across middle childhood and adolescence within samples of youths diagnosed with ADHD.

Furthermore, this study revealed that one mechanism by which the development of inattention may become associate with depressive symptoms in adolescents may involve the quality of peer relationships. Specifically, in both panels, peer relationships significantly mediated the association between inattention and depressive symptoms. In particular, both the intercept and slope of inattention significantly predicted peer relationships, which in turn was significantly associated with depressive symptoms at the final wave. This result indicates that lower levels of initial inattention symptoms and less increase or more decrease of inattention symptoms are predictive of higher levels of peer relationships. Subsequently, higher levels of peer relationships were negatively associated with depressive symptoms. The results coincide with previous studies that have found inattention as a significant predictor of youths’ peer relationships (Maedgen and Carlson, 2000; Hoza, 2007; Mikami et al., 2007), and those that have reported significant associations between peer relationships and depression (Sweeting et al., 2006; Bernaras et al., 2013; Platt et al., 2013). Youths who demonstrate inattentive behavior also show impairment in their social skills (Maedgen and Carlson, 2000; Blackman et al., 2005; Humphreys et al., 2016) and peer relationships (Hoza et al., 2005b; Mrug et al., 2007). Subsequently, compromised quality of peer relationships may serve as an environmental stressor that may contribute to high levels of depression (Platt et al., 2013 for review).

Youths learn social skills such as cooperation and conflict resolution primarily in the context of peer interaction (Rubin et al., 2006; Hoza, 2007). It has also been proposed that peer relationships may play a significant role in the development of personality (Reitz et al., 2014). Additionally, adolescence is a vulnerable period of depression (Ostrander et al., 2006), and depression in adolescence is recognized as a risk factor for future recurrent depressive episodes and other psychosocial disorders in adulthood (Rao et al., 1995). The present findings suggest that inattention trajectory spanning middle childhood and adolescence may be a predictor of adolescent depression *via* its effect on the quality of peer relationships. Our study adds to the literature in that inattention has been relatively unexplored in relation to peer relationships and depression especially in adolescence (Ahmad et al., 2020; Powell et al., 2020). Furthermore, the direct pathway from inattention trajectory to depressive symptoms, not mediated by peer relationships, was also significant, suggesting that other factors may also explain the association between inattention and depression in youths. For example, it has been suggested that family relationship (Fredrick et al., 2019), locus of control (Ostrander and Herman, 2006), or emotion regulation (Seymour et al., 2012) may also underlie the relationship between inattention and depression.

Finally, we incorporated child sex as in our conditional models, and found significant sex differences in inattention symptoms, depressive symptoms, and peer relationships. Specifically, in both Panels, boys demonstrated higher levels of inattentive symptoms compared to girls, which is consistent with the ADHD literature (Gershon and Gershon, 2002). However, it should also be noted that sex difference was insignificant for inattention symptoms at 16 years in Panel 2. There have been recent suggestions that girls’ inattention symptoms are not “fewer” than boys but more easily “overlooked” at the early developmental stages (Mowlem et al., 2019). Thus non-significant sex difference in inattention symptoms in later adolescence may arise from more identification of girls with such difficulties. In addition, our finding that girls reported higher levels of depressive symptoms than boys is coherent with ample evidence that prevalence for depression is higher for girls than boys (Petersen et al., 1991). Girls also reported higher-quality peer relationships than boys, which is also consistent with prior research that has generally found girls to display more advanced social competence than boys (Noakes and Rinaldi, 2006).

This study offers practical implications for early identification and prevention of problem behavior in adolescence. Specifically, our findings underscore the importance of targeting peer relationships and depression for youths experiencing inattention difficulties. Despite recent notion that ADHD, especially inattention symptoms, may last beyond childhood into adolescence and adulthood, relatively little is known about how inattention may interfere with individuals’ development as they grow. Although stimulant medications have been effective in alleviating ADHD symptoms (Hoza, 2007), the need for psychotherapy to address more complex difficulties such as social skills, self-esteem, and emotional problems have been underscored as well (Swanson et al., 1993). Indeed, some interventions for ADHD have already focused on promoting peer relationships to prevent emotional problems in the future (Gardner and Gerdes, 2015). However, most of those interventions have focused on elementary school children aged 5–12 (Storebo et al., 2019), but the current findings point to the potential need to extend such focus into adolescence (Hoza et al., 2005a; Storebo et al., 2019).

This study has a few limitations to note. First, participants of this study were part of a longitudinal study of general Korean youths, thus generalizing our findings to clinical populations such as patients diagnosed with ADHD might be limited. Additionally, more studies are needed to confirm our findings in ethnically and culturally more diverse backgrounds. Second, all study variables were measured using self-report questionnaires. Although self-report can provide rich information about respondents’ psychological states, it may also be susceptible to memory bias or social desirability. Especially, adolescents may be particularly susceptible to distorted responses for many reasons (Johnson and Richter, 2004), but no procedures were included in this study to control for such possibility. Therefore, in future research, it would be beneficial to minimize possible biased responses in self-report, utilize multiple methods and informants to collect data. Thirdly, the panel data used in this study lacked a measure of hyperactivity-impulsivity symptoms, and it was impossible to gauge unique role of inattention on peer relationships and depression. Incorporating both types of symptoms of ADHD would be necessary to investigate their common versus unique contribution to adolescents’ socioemotional development. Fourth, our study mostly consisted of middle-income families and thus we did not consider socioeconomic status as a correlate in conditional models. However, as socioeconomic disadvantage is known to increase the risk of children’s mental health problems including ADHD and depression (Russell et al., 2016), future research would benefit by recruiting demographically at-risk families and examine how inattention trajectories may be related to family’s socioeconomic resources. Lastly, although we used a longitudinal design and proposed a model based on prior research, it is impossible to draw conclusions regarding causality between inattention, peer relationships, and depression in adolescence. Furthermore, it should be noted that the variables of interest in this study may demonstrate bidirectional relationships. For example, although we were interested in delineating pathways from inattention to depression, depression may also compromise individuals’ cognitive functions including attention (Marazziti et al., 2010).

Despite the limitations, this study represents an initial effort to model longitudinal development of inattention symptoms in the transition to adolescence and to examine its contribution to depressive symptoms *via* peer relationships as a mediator. The findings highlight the need to track changes in inattention to early identify and intervene to children and adolescents who may be at higher risk for depression, and also to promote peer relationships as a preventive strategy to prevent depression.

## Data Availability Statement

Publicly available datasets were analyzed in this study. This data can be found here: Korean Children & Youth Panel Survey 2010 (KCYPS 2010) at https://www.nypi.re.kr/archive/mps.

## Author Contributions

SP was responsible for literature review, data analysis, and write-up. HC advised all parts of the study and write-up. Both authors contributed to the article and approved the submitted version.

## Conflict of Interest

The authors declare that the research was conducted in the absence of any commercial or financial relationships that could be construed as a potential conflict of interest.

## Publisher’s Note

All claims expressed in this article are solely those of the authors and do not necessarily represent those of their affiliated organizations, or those of the publisher, the editors and the reviewers. Any product that may be evaluated in this article, or claim that may be made by its manufacturer, is not guaranteed or endorsed by the publisher.

## Footnotes

1 Additional information about KCYPS can be found here. https://www.nypi.re.kr/archive/mps
